# Laminariales Host Does Impact Lipid Temperature Trajectories of the Fungal Endophyte *Paradendryphiella salina* (Sutherland.)

**DOI:** 10.3390/md18080379

**Published:** 2020-07-22

**Authors:** Marine Vallet, Tarik Meziane, Najet Thiney, Soizic Prado, Cédric Hubas

**Affiliations:** 1 Molécules de Comunications et Adaptation des Microorganismes (MCAM) Muséum National d’Histoire Naturelle, CNRS, 63 Rue Buffon, FR-75005 Paris, France; mvallet@ice.mpg.de (M.V.); soizic.prado@mnhn.fr (S.P.); 2 Laboratoire de Biologie des Organismes et Ecosystèmes Aquatiques (BOREA), Muséum National d’Histoire Naturelle, IRD, SU, CNRS, UA, UCN, 61 Rue Buffon, FR-75005 Paris, France; tarik.meziane@mnhn.fr (T.M.); najet.thiney@mnhn.fr (N.T.); 3 Laboratoire de Biologie des Organismes et Ecosystèmes Aquatiques (BOREA), Muséum National d’Histoire Naturelle, IRD, SU, CNRS, UA, UCN, Station Marine de Concarneau, FR-29900 Concarneau, France

**Keywords:** fatty acids, fungal endophytes, laminariales, *Paradendryphiella salina*

## Abstract

Kelps are colonized by a wide range of microbial symbionts. Among them, endophytic fungi remain poorly studied, but recent studies evidenced yet their high diversity and their central role in algal defense against various pathogens. Thus, studying the metabolic expressions of kelp endophytes under different conditions is important to have a better understanding of their impacts on host performance. In this context, fatty acid composition is essential to a given algae fitness and of interest to food web studies either to measure its nutritional quality or to infer about its contribution to consumers diets. In the present study, *Paradendryphiella salina*, a fungal endophyte was isolated from *Saccharina latissima* (L.) and *Laminaria digitata* (Hudson.) and its fatty acid composition was assessed at increasing salinity and temperature conditions. Results showed that fungal composition in terms of fatty acids displayed algal-dependent trajectories in response to temperature increase. This highlights that C18 unsaturated fatty acids are key components in the host-dependant acclimation of *P. salina* to salinity and temperature changes.

## 1. Introduction

Kelps are colonized at their surface, but also within their tissues, by a wide range of micro-organisms and thus act as hosts to species-rich assemblages of algae, animals and microbes. The associated microorganisms are responsible for spreading infectious algal diseases, protecting against fouling organisms and pathogens or producing substances that promote algal growth [1]. Among these micro-organisms, endophytic fungi remain poorly documented although recent studies evidenced their high diversity [2] and their key role in algal defence against various pathogens. Their role is still virtually unknown and there is a need to examine how environmental factors influence the relationship between the fungi and their hosts [1].

In that context, isolation of *P. salina* (Ascomycota) strains from several brown algal species has brought new insights into the complex relationships between these macroalgae and their microbiote. The observed association of the fungus to brown algae dates back to 1916 when it was first described as *Cercospora salina* [3]. Its ecological mode and habitat were described as saprophytic on seaweeds. It is extremely widespread and has then been found in many ecosystems from the tropics to mid latitudes. It occurs in salt marshes, in sediments, at the surface of living or dead algae thalli [4], sea grasses and woods and has been successfully, isolated from various plant and algal substrates at different geographical locations and climatic zones ([5,6] and references therein).

This fungus was studied for its adaptations to the abiotic and biotic parameters commonly found in its natural marine habitats. All the tested strains grew optimally on culture media with added marine salts, at pH values between 6.5 and 8.0 and at an incubation temperature of 25 ${}^{\circ }$C. It generally exhibits an increased salt optimum with increasing incubation temperature and clearly demonstrate an important phenotypic plasticity and the ability to adapt to diverse biotopes [5]. Recent studies have demonstrated that this common fungal endophyte produce bioactive pyrenocines and pyrenochaceatic acid which may confer protection to the host algae against pathogen infection [2]. Furthermore, bacterial and fungal endophytes associated to four brown algae *Ascophyllum nodosum* (L.), *Pelvetia canaliculata* (L.) *L. digitata*, and *S. latissima* produce metabolites that interfere with bacterial autoinducer-2 quorum sensing (QS), a signalling system involved in virulence and host colonization [7]. Recent results suggest that QS quenching may be linked to a novel α-hydroxy γ-butenolides produced by *P. salina* which interfere with the QS system of the pathogenic bacterial model *Pseudomonas aeruginosa* (Schroeter.) [8]. In addition, a recent study reveals the ability of *P. salina* to degrade alginate of brown algae [9].

Kelps are particularly rich in palmitic acid (16:0), palmitoleic acid (16:1n−7), oleic acid (18:1n−9), linoleic acid (18:2n−6) and arachidonic acid (20:4n−6) but composition may vary according to environmental factors especially temperature and depth [10,11]. A tendency of decreasing unsaturation towards the warmer seasons has been observed and the comparison of fatty acid profiles between *S. latissima* (L.), *Saccorhiza polyschides* (Lightfoot.), and *Laminaria ochroleuca* (Bachelot de la Pylaie.), also indicated species-specific factors [10]. In light of recent evidences of the hitherto unsuspected diversity of fungal endophytes in brown algae, it is not clear whether algal or fungal cells are responsible for previously observed changes in fatty acids composition of kelps (especially in palmitic acid together with oleic, linoleic and linolenic acids 18:3n−3) according to environmental conditions and/or species-specific factors. Most fungi are indeed very rich in C18 fatty acids [12].

These metabolites are important structural components, but also active constituents in several physiological processes. For instance, oxylipins which are key signalling molecules in stress response and immunity [13] and have important implication in fungal development and pathogen/host interactions [14] are produced enzymatically or non-enzymatically as a result of oxygenation of C18 fatty acids by free radicals and reactive oxygen species [15]. The aim of the present study was thus to explore fatty acid synthesis of a common kelp endophyte under different conditions to understand the potential role of *P. salina* on its host metabolism.

## 2. Results

### 2.1. Fatty Acid Compositions

Fatty acid compositions of *P. salina* strains LD40H and SL540T are reported in Table 1. A total of 22 fatty acids were detected. Saturated fatty acids (SFA) were lauric acid (12:0), myristic acid (14:0), pentadecylic acid (15:0), palmitic acid (16:0), margaric acid (17:0), stearic acid (18:0), arachidic acid (20:0), and behenic acid (22:0). Altogether, 16:0 and 18:0 were the most abundant SFA and contributed in average 23 ± 3 and 4 ± 1% to total fatty acids (TFA), respectively. Mono-unsaturated fatty acids (MUFA) were myristoleic acid (14:1n−5), palmitoleic acid (16:1n−7), hypogeic acid (16:1n−9), 17:1n−7, 17:1n−9, vaccenic acid (18:1n−7), oleic acid (18:1n−9), and 20:1n−9. Across all treatments, 18:1n−9 was the most abundant MUFA (28 ± 4% of TFA). Measured poly-unsaturated fatty acids (PUFA) were 16:2n−4, 16:2n−6, 17:2n−5, Linoleic acid (18:2n−6), alpha-linolenic acid (18:3n−3), and 20:2n−9. Linoleic acid was the most abundant PUFA as well as the most abundant fatty acid with 40 ± 3% of TFA.

**Figure 1:**
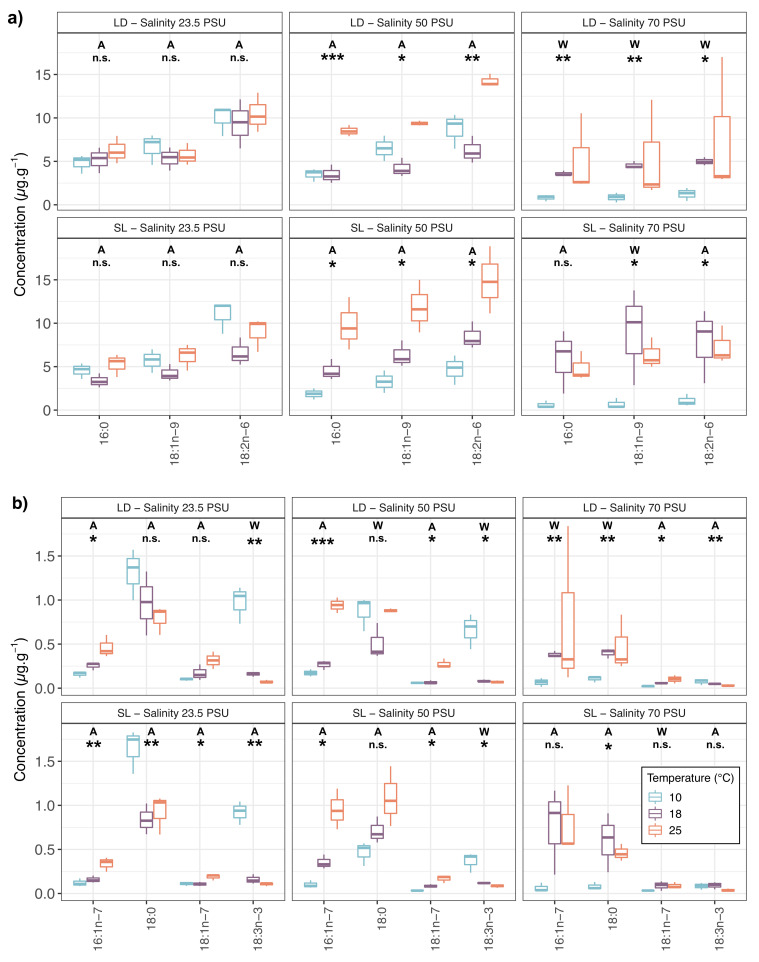
Concentrations ($\mu g\;g^{-1}$) of the 7 most abundant fatty acids. (**a**) Higher concentrations and (**b**) lower concentrations of endophytic *P. salina* isolated from *L. digitata* (LD) or *S. latissima* (SL) and grown at different salinities (23.5, 50 and 70 PSU) and temperatures (10, 18 and 25 ${}^{\circ }$C). One-way Welch ANOVA (W) or One-way ANOVA (A) has been performed depending on the result of Bartlett test: n.s. = not significant, * *p* < 0.05, ** *p* < 0.01, and *** *p* < 0.001.

Concentrations of the most abundant fatty acids were compared according to temperature and salinity treatments. Figure 1 shows the differences between temperatures for each of these fatty acids per salinity treatment and algal host. For both LD and SL temperature changes induced few modifications of fatty acids concentrations at 23.5 practical salinity units (PSU). Significant changes were observed only for minor fatty acids such as 16:1n−7 or 18:3n−3 whereas most compounds (including major *P. salina* ones) showed significant changes with temperature at 50 and 70 PSU.

### 2.2. Lipid Trajectories

**Figure 2:**
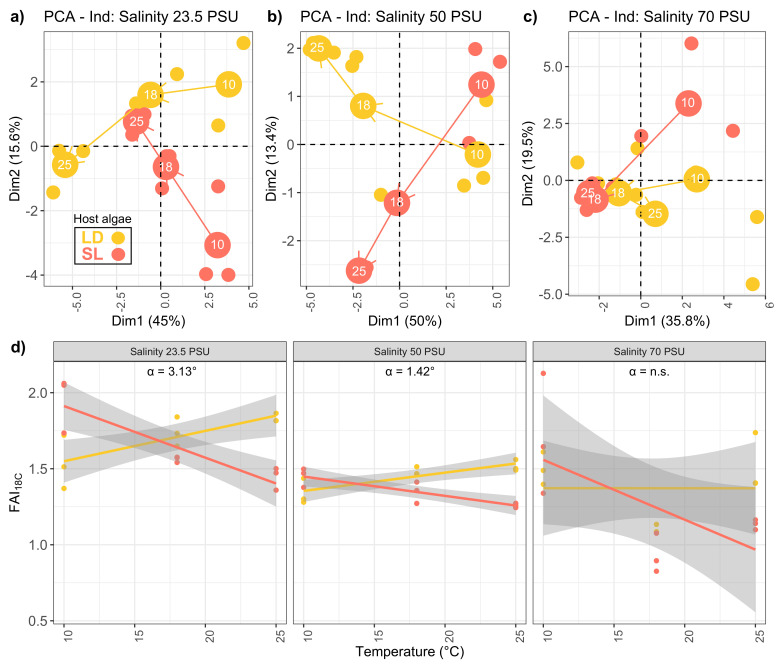
Fatty acid trajectories of endophytic *P. arenaria* isolated from *L. digitata* (LD) or *S. latissima* (SL) grown at different salinities (23.5, 50 and 70 PSU) and different temperatures (10, 18 and 25 ${}^{\circ }$C): (**a**–**c**) PCA scores. (**d**) $FAI_{18-C}$ index calculated with relative contributions (%) of major C18 fatty acids showing opposite trends in the acclimation of *P. salina* to temperature between host algae. α= angle between the two regression lines.

Principal Component Analyses (PCA) were performed using fatty acids relative proportions (in %) for each salinity treatment (Figure 2a–c). The effect of the temperature gradient on fatty acids profiles is displayed for each salinity separately. The PCA allowed to track fatty acids trajectories which corresponded to the path of gradual change in whole fatty acid composition according to T ${}^{\circ }$C. At 70 PSU, no clear trajectories were observed and the PCA ordination explained 55% of the whole inertia in comparison to 61 and 63% for 23.5 and 50 PSU (Figure 2).

Lipid trajectories showed clear opposite directions at 23.5 and 50 PSU between host-algae (LD and SL) meaning that the elevation of temperature induced opposite responses in *P. salina* according to the algal host. Further investigation of lipid trajectories showed an opposition between 18:1n−9 and 18:2n−6. A fatty acid index ($FAI_{18-C}$) was calculated using major C18 unsaturated fatty acids relative concentrations (%):

(1) $$FAI_{18-C}=\frac{\left[18:2n-6\right]}{\left[18:1n-9\right]}$$

$FAI_{18-C}$ was significantly affected by host-algae, temperature and salinity effects (Table 2) but only host-algae:temperature and temperature:salinity interactions were significant.

It also showed clear opposed linear relationships with temperature according to the algal host (Figure 2d). The index increased with temperature in LD but decreased in SL. At salinities 23.5 and 50 PSU the effect of host algae on $FAI_{18-C}$ was significant as the assumption of homogeneity of slope regression was not met (ANCOVA, interaction host-algae:temperature, F= 35.509, $df_{n}=$ 1, $df_{d}=$ 14, p= 3.49 × $10^{-5}$ and F= 34.483, $df_{n}=$ 1, $df_{d}=$ 14, p= 4.06 × $10^{-5}$ respectively). This indicated that slopes of the regression lines were significantly different. At the salinity of 70 PSU, the homogeneity of slope regression was validated (ANCOVA, interaction host-algae:temperature, F= 2.818, p= 0.115) but the effect of host algae was not significant (ANCOVA–temperature effect: F= 4.124, p= 0.06, host-algae effect: F= 0.355, p= 0.56). When regression lines were significantly different, the angle between the two regression lines was calculated (hereafter named α-value). It represented the degree of influence of host-algae in the adaptation of *P. salina* to temperature (Figure 2). This degree of influence was significantly decreasing with salinity (Supplementary Materials Figure S1).

## 3. Discussion

### 3.1. Fatty Acid Compositions of *P. salina* in Relation to Experimental Conditions

The cosmopolitan fungi *P. salina* is widely spread in all type of marine ecosystems, which clearly demonstrate its effective capacity to adapt to diverse temperature and salinity conditions [5,6]. In the present study, salinity levels (including the extreme 70 PSU) did not impact drastically total fatty acid concentrations, except a noticeable decrease at 10 ${}^{\circ }$C and 70 PSU (Supplementary Materials Figure S2), which further illustrate the capacity of the fungi to thrive at salinity conditions well beyond the growth capacity of both host-algae.

Several studies have documented the effect of environmental variables on recruitment, survival, growth, size, biomass and density of kelps, nutrient and light being key factors [16,17]. Along the NE Atlantic (Norwegian) coasts, *L. hyperborea* abundance is, for instance, primarily driven by the interaction between wave exposure and either depth or ocean currents, implying depth-specific effects of wave exposure and wave-specific effects of current speed [18]. In terms of salinity tolerance, *L. digitata* exhibit optimal growth between salinity of 23 and 31 PSU, with a strong reduction of growth at 16 PSU and high mortality below 8 PSU [1]. In a study on Artic kelps, Karsten et al. [19] showed that, on a gradient from 5 to 60 PSU, maximum effective quantum yields (a proxy for photosynthetic efficiency) were measured between 20 and 55 PSU for *L. digitata* and *S. latissima*. Thus, in the present study, while 50 PSU is already a challenging condition; 70 PSU is clearly extreme for these species. Interestingly, despite the recognised phenotypic plasticity of *P. salina* and that the fungi was able to grow at the highest studied salinity without significant loss in total lipid mass, the observed fatty acids trajectories as well as $FAI_{18-C}$ relationships were no longer observed at 70 PSU. This indicated a dynamic relationship of the fatty acid metabolism between *P. salina* and its host-algae and is emphasized by the opposed response to temperature increase between both algal host species.

### 3.2. Divergent Fatty Acid Trajectories in P. salina Revealed Adaptive Strategies to Temperature Changes

Kelp forests are found on rocky seabeds from temperate to Arctic ecosystems and many species, such as *Laminaria* sp., have an important adaptive capacity to temperature changes [1]. For instance, endemic Arctic *L. solidungula* grow at temperatures between 5 and 16 ${}^{\circ }$C, and cold-temperate NE Pacific species grow between 0 and 18 ${}^{\circ }$C with optima between 5 and 15 ${}^{\circ }$C. The growth range of cold-temperate N Atlantic species extends from 0 to 20 ${}^{\circ }$C with optima between 5 and 15 ${}^{\circ }$C while warm-temperate Atlantic species grow at up to 23–24 ${}^{\circ }$C and have slightly elevated optima [1]. The temperature gradient investigated in the present study is thus within the range of natural temperature conditions.

When submitted to this gradient, endophytic *P. salina* showed divergent fatty acids trajectories as well as $FAI_{18-C}$ relationships depending on the host. At salinities 23.5 and 50 PSU the effect of host algae on $FAI_{18-C}$ was significant. The host effect was more pronounced at 23.5 than at 50 PSU as shown by the α-value and disappeared at the extreme 70 PSU which indicated that the opposition in lipid metabolism and C18 trajectories between LD and SL are conserved throughout the salinity gradient although severe (50 PSU) and extreme (70 PSU) salinities did impact *P. salina* fatty acid metabolism.

In *L. digitata* C18 fatty acids and especially linoleic acid (18:2n−6) are essential in the response of the algae against stressful conditions such as the perception of pathogenic metabolites [20] or against grazing by specialised herbivorous species [21]. The response, in all cases, imply an oxidative stress and the activation of fatty acid oxidation cascades [22]. For instance, early events in the perception of pathogens lipopolysaccharides in this brown alga include the production of 13-hydroxyoctadecadienoic acid (13-HODE) as a result of the oxydation of 18:2n−6 by lipoxygenase activity [20]. A decrease in fatty acid occurs in *S. latissima* during the early development from gametes to gametophytes. The decrease was significant for 18:1n−9, from 45 to 30% of total fatty acids, suggesting that it might be important in the transition from storage lipids to photo-autotrophic strategies [23]. Thus, an increase in $FAI_{18-C}$ in laminariales is likely associated to the redirection of the algal lipid metabolism toward photosynthesis or defence to the detriment of storage lipids.

Homeoviscous and homeophasic adaptations, which is the process of keeping adequate membrane fluidity, as a response to temperature changes are well documented for microorganisms. Degree of unsaturation, variation in chain length, branching and cyclization of fatty acids are known adaptative strategies to enhance membrane fluidity. A considerable decrease in 18:1 and the marked increase in 18:2 or 18:3 with lower temperatures have already been observed in bacteria, fungus and yeast [24]. In the present study, any decrease in temperature is thus expected to induce an increase in $FAI_{18-C}$ as a response. However, this expected relationship was noticed only when the fungal endophyte was isolated from *S. latissima* and, intriguingly, it exhibited an opposite trend when isolated from *L. digitata*.

In absence of dedicated temperature experiments on both *L. digitata* and *S. latissima*, it is difficult to conclude on whether *P. salina* lipid metabolism was fully aligned with its host requirements. However, the observed opposed trend in lipid trajectories between the endophytic fungi of the two hosts revealed a temperature-response that was clearly host dependant.

Host species originated from separate areas (Roscoff-FR and Oban-UK for LD and SL respectively) which, despite being slightly warmer in average (2.6 ± 0.4) in Roscoff, are relatively similar in terms of sea surface temperature and salinity (SST NOAA). It is thus very likely that the two fungal strains originated from two different populations that were each adapted to their Laminariale host. Unfortunately, we do not have precise genomics information about the two endophytic strains (other than ITS barcode sequencing) to validate this hypothesis.

However, previous comparative metabolomics on the same endophytic strains, and seven additional *P. salina* isolates from various brown algae, have demonstrated a clearly divergent metabolome between algal species as well as orders (i.e., Fucales vs. Laminariales) [8]. Altogether, the present findings highlight the plasticity of the fungus to adapt to a new environment (i.e., the hosting algae). The fact that the host influenced the expression of *P. salina* metabolome may reflect epigenetic mechanisms as changes in metabolome expression [8] and lipid trajectories (this study) might be conserved across multiple generations.

## 4. Materials and Methods

### 4.1. Reagents and Chemicals

Following solvents were used: Sigma-Aldrich methanol ≥ 99.9% Cat No. 34860; ethanol ≥ 99.8% Cat No. 51976; chloroform ≥ 99% Cat No. C7559; hexane ≥ 99% Cat No. 139386. Following reagents were used for fatty acid purification and identification: BF3-methanol (boron-trifluoride methanol, Supelco^®^, CAS Number: 373-57-9, Cat No. 15716) for derivatization; Sigma-Aldrich tricosanoic acid analytical standards (Sigma-Aldrich–C23–Methyl tricosanoate, CAS Number: 2433-97-8, Cat No. T9900) as internal standard; Supelco^®^ 37 Component FAME Mix, Cat No. CRM47885n, Marine source, Cat No. 47033, and Bacterial Mix, Cat No. 47080-U for fatty acid identification.

### 4.2. Strain Isolation, Cultivation and Identification

*P. salina* strains LD40H and SL540T were previously isolated from *L. digitata* (LD) and *S. latissima* (SL) respectively. Complete isolation, cultivation and molecular identification procedures are reported in Vallet et al. [2]. Briefly, three individuals of each species were collected during spring tide and processed within two hours of collection. Algae organs of 5 ${\mathrm{cm}}^{2}$ (receptacles, thalli, stipes, fronds and holdfasts) were excised and surface-sterilized by sequential immersion in Ethanol 70% (30 s), in NaCl 0.1% (30 s) and washed three times (30 s) in sterilized sea water [25,26]. Algal segments were plated on solid media (malt extract agar, Millipore) with the internal tissues in contact with the medium and solidified with 20 g·$L^{-1}$ of purified agar. Strains corresponded to *P. salina*, a strictly marine fungus identified with ITS sequencing from all brown algal species investigated [2].

### 4.3. Experimental Design

Fungal endophytic strains LD40H and SL540T were grown on solid medium Malt Extract Agar for 21 days with 12 h photo-period under both temperature and salinity stress conditions. Several salinity concentrations were tested ranging from low ([NaCl] = 23.5 g·$L^{-1}$), elevated ([NaCl] = 50 g·$L^{-1}$) to extreme conditions ([NaCl] = 70 g·$L^{-1}$). Three incubation temperatures were also tested within the growth range of natural LD and SL population in the temperate Atlantic ocean (10 ${}^{\circ }C$, 18 ${}^{\circ }C$ and 25 ${}^{\circ }C$). Experiments were conducted in biological triplicates.

### 4.4. Fatty Acid Extraction

Fatty acid (FA) analysis was performed following the modified method of Bligh and Dyer [27] as modified by [28,29]. Before extraction, an internal standard (23:0) was added to every sample for quantification purpose (0.5 $mg\;mL^{-1}$). Lipids were extracted with a 20 min ultrasonication (sonication bath, 80 kHz, Fisherbrand™) in a mixture of distilled water, chloroform and methanol in ratio 1:1:2 (v:v:v, in mL). Lipids were concentrated under N${}_{2}$ flux, and saponified, in order to separate FA, with a mixture of NaOH (2 mol $L^{-1}$) and methanol (1:2, v:v, in mL) at 90 ${}^{\circ }C$ during 90 min. Saponification was stopped with 500 μL hydrochloric acid. Samples were then incubated with BF3-methanol at 90 ${}^{\circ }C$ during 10 min to transform free fatty acids into fatty acids methyl esters (FAME), which were isolated and kept frozen in chloroform. Just before analysis, samples were dried under N${}_{2}$ flux and transferred to hexane. One μL of the mixture was injected in a gas chromatograph (GC, Varian CP-3800 equipped with flame ionization detector), which allowed separation and quantification of FAME. Separation was performed with a Supelco^®^ OMEGAWAX 320 column (30 m × 0.32 mm i.d., 0.25 μm film thickness) with He as carrier gas. The following temperature program was used: 60 ${}^{\circ }C$ for 1 min, then raise to 150 ${}^{\circ }C$ at 40 ${}^{\circ }C$·$\min^{-1}$ (held 3 min), then raise to 240 ${}^{\circ }C$ at 3 ${}^{\circ }C$·$\min^{-1}$ (held 7 min) at 1 $mL\min^{-1}$. FAME Peaks were identified by comparison of the retention time with analytical standards. Additional identification of the samples was performed using a gas chromatograph coupled to mass spectrometer (GC-MS, Varian 450GC with Varian 220-MS). Compounds annotation was performed by comparing mass spectra with NIST 2017 library. Fatty acids were quantified using the FID detector and the internal standard (C23). Corresponding fatty acids are designated as X:Y*n*-Z, where X is the number of carbons, Y the number of double bonds and Z the position of the ultimate double bond from the terminal methyl (see [30] for additional information about naming convention).

### 4.5. Statistics

All statistical analyses were performed using R version 3.5.3 [31] and packages reshape [32], ggplot2 [32], rstatix [33], ade4 [34], factoextra [35] and cowplot [36] for data processing and visualisation. Raw GC-FID text file data together with in-house R script for data processing, univariate and multivariate statistics as well as figures are available at github repository: https://github.com/Hubas-prog/Paradendryphiella_traject.

#### 4.5.1. Univariate

Comparisons in fatty acids concentrations were performed using Analysis of Variances (ANOVA) after prior verification of the normality of the residuals (Shapiro test) and equality of variance (Bartlett test). When Bartlett test indicated that homoscedasticity was not met, one-way Welch’s ANOVA (for unequal variances) was performed otherwise, classical one-way ANOVA was used.

Analysis of covariance (ANCOVA) was performed to compare $FAI_{18-C}$ index between temperatures within each salinity treatments. Linearity was inspected visually and homogeneity of regression slopes was checked by testing the interaction between temperature and host-algae. Regression lines were considered different if slopes were different. When slopes were not significantly different, the ANCOVA was performed by checking the presence of outliers and by removing the interaction effect to adjust the ANCOVA model.

#### 4.5.2. Multivariate

Lipid trajectories were calculated using Principal Component Analysis (PCA). PCA allowed to track fatty acids changes in relative proportion in the whole fatty acid profile rather than in a given compound. The aim was to check whether fatty acid trajectories were convergent, divergent or alike in response to temperature increase. Trajectories were studied by comparing PCA scores (i.e., individuals) between host-algae and within each salinity treatment. PCA loadings (i.e., variables) was inspected visually to detect which fatty acids were responsible for lipid trajectories. Univariate staticitics were then performed as described above to validate any changes.

## Figures and Tables

**Table 1:** Fatty acids concentrations ($\mu g\;g^{-1}$) in the extracts of endophytic *P. salina* isolated from *L. digitata* (LD) or *S. latissima* (SL) and grown at different salinities (S1, S2 and S3 at 23.5, 50 and 70 PSU respectively) and temperatures (T10, T18 and T25 at 10, 18 and 25 ${}^{\circ }$C respectively). <dl indicates the signal was below detection limit.

			12:0		14:0		14:1n-5		15:0		16:0		16:1n-7		16:1n-9		16:2n-4	
Host algae	Temperature	Salinity	mean	sd	mean	sd	mean	sd	mean	sd	mean	sd	mean	sd	mean	sd	mean	sd
LD	T10	S1	0.02	0.022	0.04	0.007	0.01	0.001	<dl	<dl	4.79	1.069	0.16	0.035	0.03	0.005	0.01	0.003
SL	T10	S1	<dl	<dl	0.03	0.005	<dl	<dl	0.01	0.001	4.55	0.899	0.12	0.045	0.03	0.008	0.01	0.002
LD	T18	S1	0.01	0.010	0.03	0.011	0.01	0.000	0.01	0.002	5.2	1.472	0.25	0.044	0.04	0.008	0.02	0.004
SL	T18	S1	<dl	<dl	0.02	0.003	<dl	<dl	0.01	0.002	3.36	0.810	0.16	0.040	0.03	0.008	0.01	0.001
LD	T25	S1	0.01	0.005	0.03	0.004	0.02	0.015	0.02	0.005	6.24	1.586	0.46	0.126	0.06	0.016	0.03	0.009
SL	T25	S1	<dl	<dl	0.03	0.005	0.01	0.003	0.01	0.003	5.26	1.311	0.34	0.081	0.04	0.008	0.02	0.004
LD	T10	S2	0.01	0.006	0.03	0.007	<dl	<dl	0.01	0.001	3.49	0.741	0.17	0.040	0.02	0.005	0.01	0.002
SL	T10	S2	0.01	0.011	0.02	0.001	<dl	<dl	<dl	<dl	1.87	0.640	0.1	0.044	0.01	0.005	<dl	<dl
LD	T18	S2	0.01	0.017	0.03	0.004	0.01	0.003	0.01	0.003	3.47	1.061	0.27	0.053	0.04	0.008	0.01	0.004
SL	T18	S2	0.01	0.001	0.04	0.009	0.01	0.001	0.02	0.004	4.55	1.194	0.35	0.081	0.04	0.008	0.02	0.005
LD	T25	S2	0.01	0.002	0.08	0.030	0.02	0.010	0.02	0.005	8.5	0.643	0.94	0.088	0.09	0.008	0.07	0.004
SL	T25	S2	<dl	<dl	0.09	0.031	0.02	0.003	0.03	0.007	9.8	3.034	0.95	0.230	0.08	0.014	0.05	0.014
LD	T10	S3	0.01	0.002	0.01	0.004	<dl	<dl	<dl	<dl	0.82	0.373	0.07	0.049	<dl	<dl	<dl	<dl
SL	T10	S3	<dl	<dl	0.01	0.004	<dl	<dl	<dl	<dl	0.62	0.418	0.06	0.051	<dl	<dl	<dl	<dl
LD	T18	S3	<dl	<dl	0.04	0.004	<dl	<dl	0.01	0.001	3.6	0.277	0.38	0.037	0.01	0.003	0.02	0.002
SL	T18	S3	<dl	<dl	0.07	0.044	<dl	<dl	0.02	0.013	5.91	3.655	0.76	0.493	0.03	0.020	0.03	0.017
LD	T25	S3	0.03	0.037	0.07	0.038	<dl	<dl	0.01	0.005	5.19	4.629	0.76	0.937	0.02	0.021	0.06	0.081
SL	T25	S3	<dl	<dl	0.07	0.036	<dl	<dl	0.02	0.008	4.87	1.677	0.78	0.383	0.03	0.008	0.04	0.022
16:2n-6		17:0		17:1n-7		17:1n-9		17:2n-5		18:0		18:1n-7		18:1n-9	
Host algae	Temperature	Salinity	mean	sd	mean	sd	mean	sd	mean	sd	mean	sd	mean	sd	mean	sd	mean	sd
LD	T10	S1	0.01	0.002	0.03	0.006	0.01	0.003	<dl	<dl	<dl	<dl	1.31	0.291	0.1	0.015	6.6	1.774
SL	T10	S1	0.01	0.010	0.04	0.002	0.01	0.001	0.01	0.008	<dl	<dl	1.64	0.251	0.11	0.022	5.71	1.359
LD	T18	S1	0.02	0.003	0.03	0.006	<dl	<dl	<dl	<dl	<dl	<dl	0.97	0.363	0.17	0.090	5.34	1.333
SL	T18	S1	0.01	0.004	0.03	0.008	0.01	0.001	<dl	<dl	<dl	<dl	0.84	0.174	0.11	0.023	4.21	0.980
LD	T25	S1	0.04	0.014	0.04	0.016	0.01	0.002	<dl	<dl	0.01	0.002	0.79	0.161	0.32	0.098	5.72	1.262
SL	T25	S1	0.02	0.004	0.03	0.007	0.01	0.001	<dl	<dl	<dl	<dl	0.93	0.224	0.19	0.034	6.23	1.515
LD	T10	S2	<dl	<dl	0.01	0.001	<dl	<dl	<dl	<dl	<dl	<dl	0.87	0.195	0.06	0.009	6.5	1.456
SL	T10	S2	<dl	<dl	0.01	0.007	<dl	<dl	<dl	<dl	<dl	<dl	0.47	0.135	0.04	0.007	3.27	1.289
SL	T18	S2	0.01	0.004	0.02	0.005	0.01	0.001	<dl	<dl	<dl	<dl	0.71	0.150	0.08	0.019	6.33	1.516
LD	T25	S2	0.05	0.008	0.03	0.008	0.01	0.001	0.01	0.001	0.01	0.000	0.88	0.021	0.27	0.054	9.38	0.268
SL	T25	S2	0.02	0.003	0.02	0.003	0.01	0.001	<dl	<dl	<dl	<dl	1.09	0.341	0.17	0.045	11.85	3.027
LD	T10	S3	<dl	<dl	<dl	<dl	<dl	<dl	<dl	<dl	<dl	<dl	0.11	0.035	0.02	0.011	0.85	0.551
SL	T10	S3	<dl	<dl	<dl	<dl	<dl	<dl	<dl	<dl	<dl	<dl	0.08	0.043	0.03	0.004	0.7	0.599
LD	T18	S3	<dl	<dl	0.01	0.002	<dl	<dl	<dl	<dl	<dl	<dl	0.4	0.056	0.06	0.007	4.53	0.443
SL	T18	S3	<dl	<dl	0.01	0.008	0.01	0.005	<dl	<dl	<dl	<dl	0.6	0.336	0.09	0.054	8.92	5.547
LD	T25	S3	<dl	<dl	0.01	0.002	0.01	0.005	<dl	<dl	<dl	<dl	0.47	0.318	0.1	0.046	5.38	5.823
SL	T25	S3	<dl	<dl	0.01	0.002	0.01	0.002	<dl	<dl	<dl	<dl	0.46	0.096	0.09	0.037	6.37	1.769
			18:2n-6		18:3n-3		20:0		20:1n-9		20:2n-9		22:0				
Host algae	Temperature	Salinity	mean	sd	mean	sd	mean	sd	mean	sd	mean	sd	mean	sd			
LD	T10	S1	9.92	1.743	0.97	0.214	0.04	0.009	0.01	0.005	0.03	0.005	0.02	0.003			
SL	T10	S1	10.98	1.901	0.92	0.133	0.03	0.006	0.01	0.001	0.04	0.005	0.02	0.002			
LD	T18	S1	9.38	2.822	0.16	0.027	0.03	0.015	0.01	0.002	0.04	0.015	0.02	0.005			
SL	T18	S1	6.59	1.594	0.16	0.054	0.02	0.003	0.01	0.002	0.02	0.004	0.01	0.001			
LD	T25	S1	10.48	2.265	0.07	0.018	0.02	0.004	0.01	0.005	0.07	0.017	0.02	0.002			
SL	T25	S1	8.95	1.945	0.11	0.019	0.02	0.005	0.01	0.001	0.04	0.022	0.02	0.004			
LD	T10	S2	8.71	2.021	0.66	0.199	0.04	0.008	0.01	0.003	0.05	0.023	0.01	0.005			
SL	T10	S2	4.7	1.691	0.37	0.113	0.02	0.007	0.01	0.002	0.03	0.019	0.01	0.008			
LD	T18	S2	6.23	1.565	0.08	0.015	0.02	0.008	<dl	0.002	0.05	0.026	0.01	0.003			
SL	T18	S2	8.46	1.561	0.12	0.011	0.03	0.007	0.01	0.003	0.03	0.012	0.02	0.003			
LD	T25	S2	14.24	0.745	0.07	0.017	0.04	0.005	0.01	0.003	0.05	0.019	0.02	0.002			
SL	T25	S2	14.92	3.861	0.09	0.019	0.05	0.017	0.01	0.004	0.05	0.017	0.04	0.011			
LD	T10	S3	1.24	0.743	0.07	0.036	<dl	<dl	<dl	<dl	0.01	0.004	<dl	<dl			
SL	T10	S3	1.08	0.697	0.08	0.038	<dl	<dl	<dl	<dl	<dl	<dl	<dl	<dl			
LD	T18	S3	4.99	0.463	0.05	0.004	0.02	0.003	0.01	0.002	0.02	0.005	0.01	0.002			
SL	T18	S3	7.84	4.272	0.09	0.041	0.03	0.015	0.01	0.005	0.02	0.006	0.01	0.005			
LD	T25	S3	7.75	8.020	0.03	0.011	0.02	0.020	0.01	0.006	0.03	0.020	0.02	0.009			
SL	T25	S3	7.25	2.175	0.04	0.017	0.02	0.004	0.01	0.003	0.02	0.004	0.01	0.002			

**Table 2:** Thee-way analysis of variance (ANOVA) of $FAI_{18-C}$ index as a function of host-algae, temperature and salinity. Normality assumption by group was tested using Shapiro–Wilk. In total, 14 out of 18 groups showed p> 0.05. Homogeneity of variance was tested using a Levene’s test ($df_{1}=$ 17, $df_{2}=$ 36, statistic = 1.19, p= 0.321). n.s. = not significant, * *p* < 0.05, and *** *p* < 0.001.

	df	Sum of Square	Mean Squares	*F*	*p*-Value	
Host algae (host)	1	0.1020	0.1020	5.661	0.022774	*
Temperature (temp)	2	0.4321	0.2160	11.985	0.000102	***
Salinity (sal)	2	1.2981	0.6491	36.009	2.57 × $10^{-9}$	***
host:temp interaction	2	0.8079	0.4040	22.411	4.76 × $10^{-7}$	***
host:sal interaction	2	0.0098	0.0049	0.272	0.763277	n.s.
temp:sal interaction	4	0.6356	0.1589	8.815	4.55 × $10^{-5}$	***
host:temp:sal interaction	4	0.0804	0.0201	1.115	0.364467	n.s.
Residuals	36	0.6489	0.0180			

## Supplementary Materials

The following are available online at https://www.mdpi.com/1660-3397/18/8/379/s1, Figure S1: α value as a function of salinity; Figure S2: Total fatty acid concentrations of *P. salina*.

## Abbreviations

The following abbreviations are used in this manuscript:

LD	*Laminaria digitata*
MPB	Microphytobenthos
MUFA	Monounsaturated fatty acid
PUFA	Polyunsaturated fatty acid
QS	Quorum Sensing
SFA	Saturated fatty acid
SL	*Saccharina latissima*
TFA	Total Fatty Acids
PSU	practical salinity units
ANOVA	analysis of variance
PCA	principal component analyses
FAME	Fatty acid methyl esters
NIST	National Institute of Standards and Technology
FID	flame ionization detector
