# Supplementary material for: Laminariales Host Does Impact Lipid Temperature Trajectories of the Fungal Endophyte Paradendryphiella salina (Sutherland.)
Source: Mar Drugs. 2020 Jul 22;18(8):379. doi: 10.3390/md18080379 (PMC7460085; doi:10.3390/md18080379)
Supplement: Supplementary file 1 [file marinedrugs-18-00379-s001.pdf]

marine drugs

Supplementary File :

Laminariales Host Does Impact Lipid Temperature Trajectories of the Fungal Endophyte Paradendryphiella salina (Sutherland.)

Marine Vallet [1,†,‡] , Tarik Meziane [2] , Najet Thiney [2], Soizic Prado [1] and Cédric Hubas [3,‡,_]

[1] Molécules de Comunications et Adaptation des Microorganismes (MCAM) Muséum National d'Histoire Naturelle, CNRS, 63 rue Buffon, FR-75005 Paris, France; mvallet@ice.mpg.de (M.V.); soizic.prado@mnhn.fr (S.P.)

[2] Laboratoire de Biologie des Organismes et Ecosystèmes Aquatiques (BOREA), Muséum national d'Histoire naturelle, IRD, SU, CNRS, UA, UCN, 61 rue Buffon, FR-75005 Paris, France; tarik.meziane@mnhn.fr (T.M.); najet.thiney@mnhn.fr (N.T.)

[3] Laboratoire de Biologie des Organismes et Ecosystèmes Aquatiques (BOREA), Muséum national d'Histoire naturelle, IRD, SU, CNRS, UA, UCN, Station Marine de Concarneau, FR-29900 Concarneau, France

* Corresponding author: cedric.hubas@mnhn.fr

† Current address: Max Planck Institute for Chemical Ecology, Max Planck Fellow Group Plankton Community Interaction, Friedrich-Schiller-Universität,Lessingstr. 8, D-07743 Jena, Germany

‡ These authors contributed equally to this work

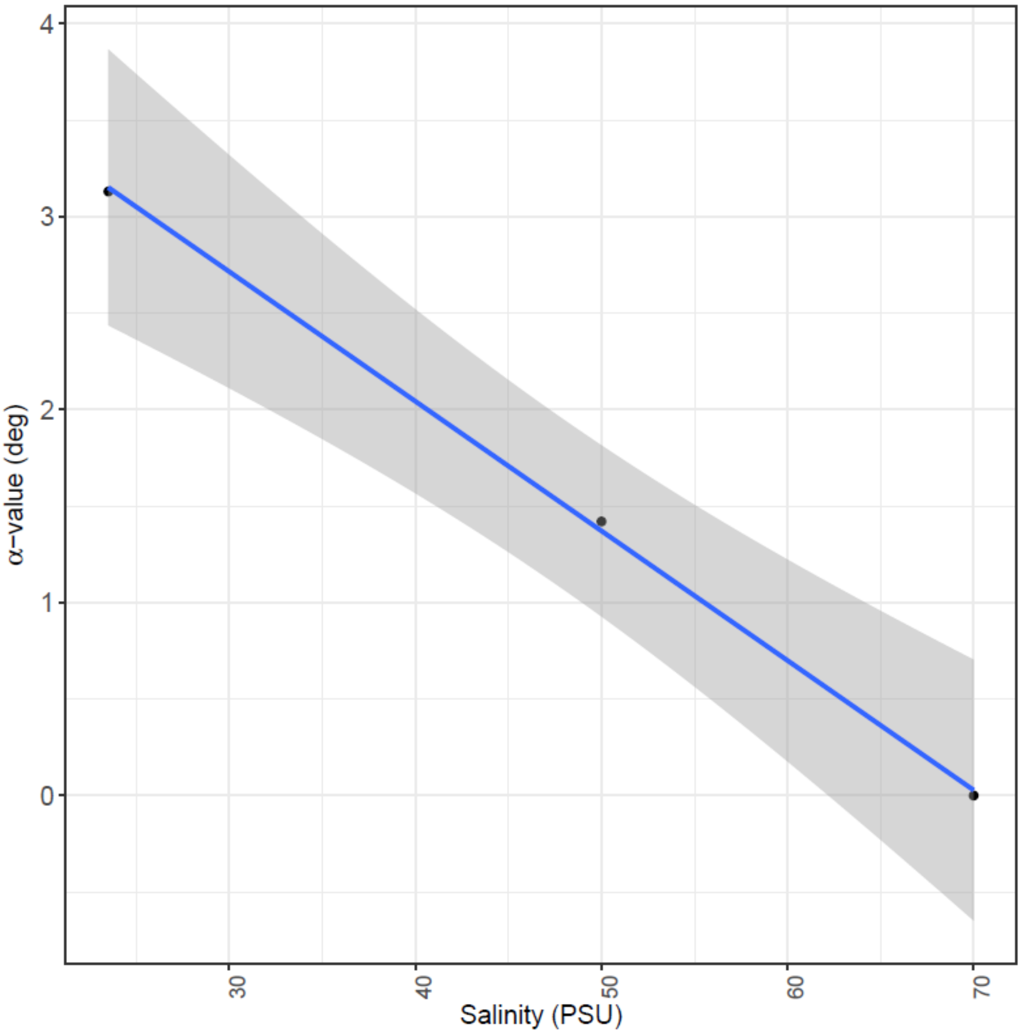

Figure S1. Angle *a* (in degree) between $FAI_{18\text{-}C}$ regression curves as a function of salinity. *a*-value. represents the degree of influence of host-algae in the adaptation of P. salina to temperature. Adjusted $R_2 = 0.9985$, $F = 1362$ on 1 and 1 d f , $p = 0.01725$. The grey area represents the confidence interval.

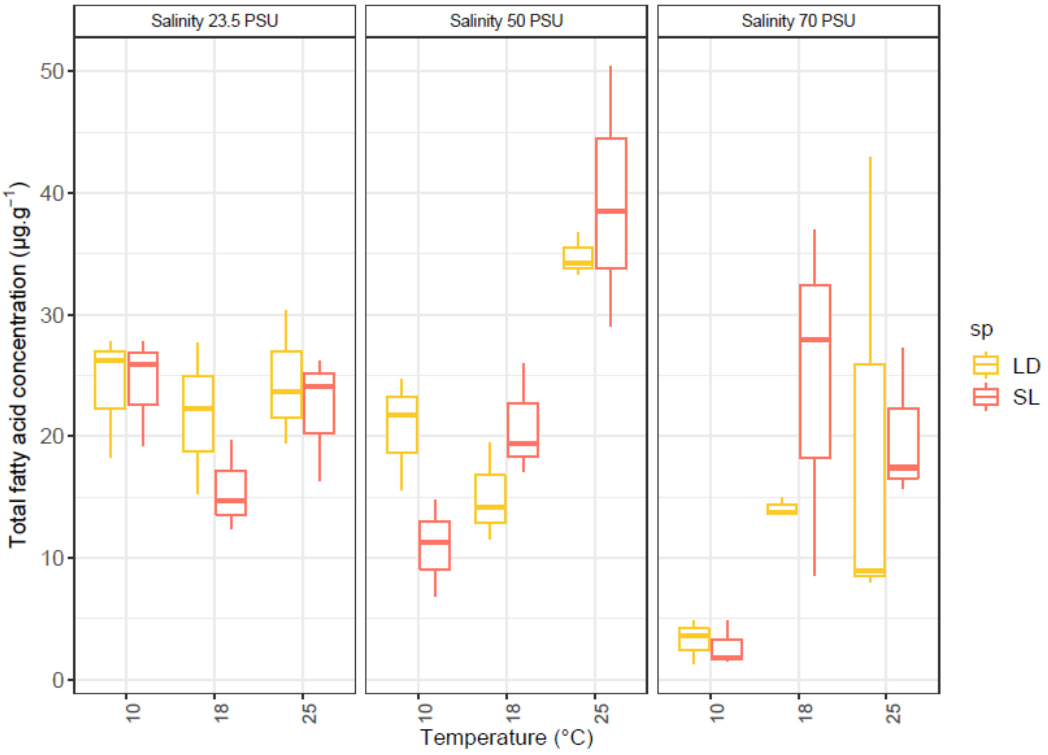

Figure S2. Total fatty acid concentrations of P. salina isolated from L. digitata (LD) or S. latissima (SL).grown at different salinities (23.5, 50 and 70 PSU) and different temperatures (10, 18 and 25 °C):
